# Genetic basis and identification of candidate genes for wooden breast and white striping in commercial broiler chickens

**DOI:** 10.1038/s41598-021-86176-4

**Published:** 2021-03-24

**Authors:** Juniper A. Lake, Jack C. M. Dekkers, Behnam Abasht

**Affiliations:** 1 Center for Bioinformatics and Computational Biology, University of Delaware, Newark, DE USA; 2 Department of Animal and Food Sciences, University of Delaware, Newark, DE USA; 3 Department of Animal Science, Iowa State University, Ames, IA USA

**Keywords:** Genetics, Agricultural genetics

## Abstract

Wooden breast (WB) and white striping (WS) are highly prevalent and economically damaging muscle disorders of modern commercial broiler chickens characterized respectively by palpable firmness and fatty white striations running parallel to the muscle fiber. High feed efficiency and rapid growth, especially of the breast muscle, are believed to contribute to development of such muscle defects; however, their etiology remains poorly understood. To gain insight into the genetic basis of these myopathies, a genome-wide association study was conducted using a commercial crossbred broiler population (n = 1193). Heritability was estimated at 0.5 for WB and WS with high genetic correlation between them (0.88). GWAS revealed 28 quantitative trait loci (QTL) on five chromosomes for WB and 6 QTL on one chromosome for WS, with the majority of QTL for both myopathies located in a ~ 8 Mb region of chromosome 5. This region has highly conserved synteny with a portion of human chromosome 11 containing a cluster of imprinted genes associated with growth and metabolic disorders such as type 2 diabetes and Beckwith-Wiedemann syndrome. Candidate genes include *potassium voltage-gated channel subfamily Q member 1* (*KCNQ1*), involved in insulin secretion and cardiac electrical activity, *lymphocyte-specific protein 1* (*LSP1*), involved in inflammation and immune response.

## Introduction

The modern commercial broiler chicken embodies remarkable gains in the economics of meat production realized through intensive breeding programs, optimized nutrition, and enhanced management practices. Compared to the 1950s, modern broilers can be raised to approximately the same market weight in close to half the time using substantially less feed and with substantially higher breast muscle yield^1–3^. However, the financial gains and increased production capacity associated with improvements to production traits are threatened by the concurrent global emergence of numerous muscle disorders that severely affect meat quality and may also impact animal welfare^4–6^. Wooden breast and white striping, often co-occurring and believed to be part of the same disease spectrum^7,8^, are two such myopathies, which together represent the breast muscle defects with the highest prevalence and greatest economic burden.

First described in the literature in 2014^4^, wooden breast manifests as palpably firm and discolored pectoralis major with subcutaneous and fascial edema, petechial hemorrhages, and spongy areas with disintegrating myofiber bundles. Birds affected by wooden breast frequently show signs of white striping as well, which is macroscopically characterized by white fatty striations running parallel to the muscle fibers and presents with similar histological lesions as wooden breast, including myodegeneration with regeneration, necrosis, lymphocyte and macrophage infiltration, fibrosis, and lipidosis^4,9^. These muscle disorders present an exceptional challenge to producers, as dietary or management strategies against them often fail to improve meat quality^10,11^ or lack viability due to impaired live performance^12^ or cost-prohibitive inputs. The tight association between breast muscle disorders and economic traits such as feed efficiency and breast muscle yield suggests that successful mitigation of meat quality defects without simultaneous compromise to desirable traits will require an understanding of the genetic basis of these myopathies and selection against their causal variants.

Several hypotheses exist regarding the underlying causes of wooden breast and white striping. Some implicate the rapid growth of the pectoralis major and relative vascular deficiency for creating a buildup of waste products and hypoxic conditions^13,14^ in the breast muscle, while others suggest shared etiologic underpinnings with type 2 diabetes and other metabolic disorders in mammals^15,16^. However, current knowledge regarding the genetic basis of wooden breast and white striping is extremely limited and somewhat conflicting. Bailey et al.^17^ estimated heritability (h^2^) of wooden breast and white striping in a purebred commercial broiler line to be low – 0.07 for wooden breast and 0.25 for white striping—but also demonstrated a dramatic 18.4% reduction in wooden breast incidence after only 2 years of genetic selection against breast muscle myopathies. Another study of two broiler lines divergently selected for ultimate pH of the pectoralis major estimated heritability (h^2^) of white striping to be 0.65^18^. The only genome-wide association study of white striping was performed on a similar population and found no markers with genome-wide significance^19^, while the genetic architecture of wooden breast currently remains unexplored.

Our poor understanding of these traits at the genetic level precludes our ability to adequately mitigate their effects through either broiler breeding or management. Therefore, the aim of this study is to estimate genetic parameters for wooden breast, white striping, and two body weight traits in a hybrid commercial broiler population and to identify quantitative trait loci (QTLs) and candidate genes to elucidate potential molecular mechanisms contributing to myopathy development.

## Results and discussion

### Trait statistics and genetic parameter estimates

Of the 1,194 progeny that were genotyped for this study, only one bird did not meet sequence filter criteria and was excluded from all analyses. Sex chromosomes were used to confirm each bird’s sex, and found that 12 birds were mis-gendered at necropsy. The sex-specific and overall distributions of wooden breast and white striping scores for the 1,193 birds that passed filter criteria can be found in Supplementary Table S1. The prevalence of wooden breast, i.e. the proportion of birds with a wooden breast score greater than 0, was approximately 79%, although the scoring system implemented in this study was relatively sensitive to mild signs of the myopathy compared to other studies^10,12^. Meat quality is not substantially affected in birds with scores of 1 or 2, and severe wooden breast (score of 4) was detected only in 2.1% of chickens in our study (Supplementary Table S1). The prevalence of white striping was similarly high, at approximately 80%. Compared to males, female birds exhibited a lower prevalence of wooden breast (71% vs. 87%; *p*-value < 0.001) and white striping (73% vs. 87%; *p*-value < 0.001).

Descriptive statistics, heritability estimates, and variance components of the traits are summarized in Table 1. In our population, the wooden breast phenotype exhibited substantially higher heritability (*h*^2^ = 0.49) than indicated by previous work, which estimated heritability between 0.1 and 0.24^20^. Additionally, we found the heritability of white striping (*h*^2^ = 0.50) to lie between previous estimates ranging from 0.18^20^ to 0.65^18^. Variability among these studies is not surprising, as heritability is inherently specific to the population and environment in which it is estimated. For example, the lower estimates for heritability of wooden breast and white striping were for purebred commercial broiler lines that had relatively low incidence rates of myopathy—0.16% to 0.39% for wooden breast; 14.46% to 49.6% for white striping—compared to other hybrid commercial broiler populations^20^. Moderate heritability for wooden breast and white striping indicates that both genetic and environmental factors exert strong influences on phenotypic differences for these traits, which is consistent with studies that have demonstrated the ability to reduce severity and incidence of breast muscle myopathies through both genetic selection^17^ and manipulation of dietary energy^12,21^.

**Table 1:** Trait statistics and estimates (± SE) of heritability and residual variance from univariate analyses of wooden breast, white striping, and body weight at 13 days and at 7 weeks of age.

Trait	Number of samples	Mean	SD	Heritability	Residual variance
Wooden Breast	1193	1.57	1.06	0.49 ± 0.06	0.51 ± 0.05
White Striping	1193	1.14	0.77	0.50 ± 0.06	0.28 ± 0.03
Body Weight 13d (g)	1193	354	57	0.36 ± 0.06	1,548 ± 124
Body Weight 7wk (g)	1193	3496	449	0.40 ± 0.06	45,085 ± 3741

Wooden breast was scored on a 5-point scale from 0-Normal to 4-Severe. White striping was scored on a 4-point scale from 0-Normal to 3-Severe.

Our analysis found genetic correlations (Table 2) of wooden breast and white striping with body weight at 13 days (− 0.04 and 0.15 respectively) and body weight at 7 weeks (0.16 and 0.09 respectively) to be low, while the estimate of the genetic correlation between the two myopathies was high (0.88). The latter reinforces an existing hypothesis that these two traits are related to each other and may be variations of the same disorder^7,8^. Note that low genetic correlation between the muscle disorders and body weight traits does not signify low genetic correlations of muscle disorders with other performance traits that were not assessed in this study, such as breast muscle yield and feed conversion ratio.

**Table 2:** Estimates (± SE) of phenotypic (above diagonal) and genetic (below diagonal) correlations among wooden breast, white striping, and body weight at 13 days and at 7 weeks of age based on bivariate analyses.

	Wooden breast	White striping	Body weight 13d	Body weight 7wk
Wooden breast		0.65 ± 0.02	0.12 ± 0.03	0.25 ± 0.03
White striping	0.88 ± 0.04		0.17 ± 0.03	0.19 ± 0.03
Body weight 13d	− 0.04 ± 0.12	0.15 ± 0.12		0.49 ± 0.03
Body weight 7wk	0.16 ± 0.11	0.09 ± 0.11	0.54 ± 0.09	

### Sequence and SNP statistics

Excluding the single sample that did not meet filter criteria, sequencing produced an average of 5.88 million reads per sample, with an average mapping rate of 77.3%. A total of 199,957 SNPs were retained after locus filtering, consisting of 195,617 autosomal SNPs and 4340 SNPs on the Z chromosome. The average sequence depth for each genotyped SNP was 10.8, with 97.9% of all genotype calls supported by at least 1 read. The average minor allele frequency was 0.24. Filter criteria implemented in this study were substantially more stringent than in previous studies utilizing low-depth genotyping-by-sequencing methods^22^ in order to identify QTLs with greater confidence.

### Linkage disequilibrium

Linkage disequilibrium (r^2^) in the population decayed at an extremely rapid rate (Supplementary Table S2), dropping below 0.2 at a distance of 2092 base pairs (bp) on macro-chromosomes, at 1410 bp on intermediate chromosomes, and at 1046 bp on micro-chromosomes. This value was substantially higher on the Z chromosome, at about 78 kb, because this chromosome is subjected to lower recombination frequency and a smaller effective population size. Based on D_0.2_ estimates for each chromosome group, SNPs in the current dataset encompassed 27.5% of the genome (autosomes and Z chromosome), with chromosome-specific capture rates ranging from 84.6% on the Z chromosome to 3.33% on chromosome 31 (Supplementary Table S3).

### GWAS results for wooden breast

**Figure 1:**
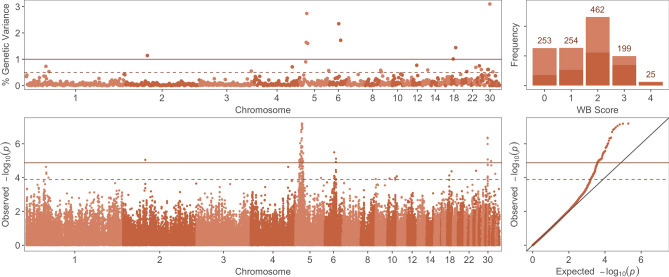
Genome-wide association results for wooden breast (WB) score using multi-marker (BayesB) and single-SNP (mixed linear model) analyses. (Top Left) Percentage of genetic variance explained by 1-Mb regions across the genome for WB score. (Top Right) Distribution of WB scores across progeny used in genome-wide association analyses; dark red = male, light red = female. (Bottom Left) Manhattan plot of single-SNP results showing the − log10(*p*-value) of SNPs ordered by chromosome and position. (Bottom Right) Quantile–quantile plot of *p*-values from single-SNP results of WB score. Solid and dashed lines indicate significant and suggestive thresholds, respectively, for each model.

Single-SNP analysis of the genetic basis of wooden breast identified 51 SNPs that were significantly associated and an additional 71 SNPs that were suggestively associated with wooden breast score (Fig. 1; Supplementary Table S4). These loci are located across 11 chromosomes of the *Gallus gallus* genome, including (GGA) 1, 2, 4, 5, 6, 9, 10, 18, 24, 30, and 32; however, the majority of significant markers (42 SNPs, 82%) and significant/suggestive markers combined (84 SNPs, 69%) were confined to a region on GGA5 from 8.1 to 16.4 Mb. A total of 28 QTLs on chromosomes (GGA) 2, 5, 6, 30, and 32 were identified from significant SNPs that were clustered based on LD (Table 3). Bayesian multi-marker regression corroborated results from single-SNP analysis, detecting 9 1-Mb windows, on chromosomes (GGA) 2, 5, 6, 18, and 30, that each explained more than 1% of genetic variance for the trait (Table 4). Together, these 9 windows explained 16.7% of the genetic variance for wooden breast. Three windows on GGA5, from 13.0 to 17.0 Mb, explained a total of 6% of genetic variance, although the single highest-ranking window in this analysis was at the beginning of GGA30, which explained 3.1% of genetic variance. Although no significant QTLs were identified for sex-specific analyses of wooden breast, some markers exceeded the suggestive threshold in female birds and are listed in Supplementary Table S5. Genomic windows with suggestive significance in the BayesB analyses are listed in Supplementary Table S6 for all traits.

**Table 3:** Wooden Breast QTL regions containing SNPs with FDR adjusted *p*-values less than 0.05.

QTL region				Top SNP in QTL region						"1">
Chromosome	Start (bp)	End (bp)	Number of significant SNPs	Position (bp)	Alleles (Effect/ Alternative)	Effect allele frequency	Effect size estimate	p-value	FDR	Candidate genes
2	–	–	1	45199243	T/C	0.473	0.211	8.91e−06	0.043	–
5	8327574	8393723	3	8393723	A/T	0.228	0.289	9.17e−06	0.043	*MICAL2* *DKK3*
5	–	–	1	9797353	A/G	0.240	0.305	4.10e−06	0.031	*NRIP3*
5	9975375	10239083	2	9975375	T/C	0.232	0.325	9.28e−07	0.014	*DENND2B* *RPL27A*
5	–	–	1	10415181	T/A	0.390	0.246	7.83e−06	0.043	*–*
5	–	–	1	10675242	T/A	0.181	0.291	1.10e−05	0.046	*PSMA1*
5	11903835	11903902	4	11903836	G/A	0.235	−0.197	8.68e−06	0.043	*–*
5	–	–	1	11913861	A/G	0.163	−0.305	1.15e−05	0.046	*–*
5	–	–	1	11924135	T/C	0.255	0.300	1.76e−06	0.019	*–*
5	12196653	12196751	2	12196653	T/C	0.232	−0.352	1.50e−07	0.004	*USH1C*
5	12310175	12310343	7	12310234	T/C	0.244	−0.270	6.52e−07	0.011	*–*
5	13374229	13429787	2	13429787	T/C	0.240	−0.313	4.75e−07	0.008	*KCNQ1* *SLC22A18* *CDKN1C*
5	13449328	13449379	3	13449367	T/C	0.369	−0.281	6.38e−08	0.004	*KCNQ1*
5	–	–	1	13508017	C/A	0.245	0.301	7.27e−06	0.043	*KCNQ1*
5	–	–	1	13899194	G/C	0.255	−0.284	1.15e−05	0.046	*–*
5	14280580	14280613	2	14280613	T/C	0.335	0.311	6.47e−08	0.004	*LSP1*
5	14295515	14308280	4	14301905	G/C	0.180	−0.307	1.44e−06	0.018	*TNNI2* *SYT8* *ENSGALG00000006608*
5	14321077	14321102	2	14321077	G/A	0.433	0.280	1.32e−07	0.004	*CTSD* *ENSGALG00000006608*
5	–	–	1	14529930	T/C	0.286	0.065	7.87e−06	0.043	*BRSK1* *ENSGALG00000006608*
5	–	–	1	16063707	T/C	0.174	0.319	1.06e−05	0.046	*ENSGALG00000044313* *PHRF1*
5	16219365	16219383	2	16219365	A/G	0.213	−0.292	3.66e−06	0.029	*HRAS*
6	–	–	1	19640631	T/G	0.389	0.343	3.21e−06	0.027	*–*
6	–	–	1	23100280	A/G	0.303	0.294	1.20e−05	0.047	*GOT1* *CNNM1*
6	23389752	23391597	3	23389752	A/G	0.119	0.368	7.59e−06	0.043	*LCOR*
30	–	–	1	90012	C/T	0.292	−0.249	4.34e−07	0.008	*DNM2* *QTRT1*
30	96694	97362	2	97362	A/G	0.196	−0.283	4.73e−07	0.008	*DNM2*
30	–	–	1	393145	A/G	0.275	0.240	8.50e−06	0.043	*CNN1* *ZNF653* *ECSIT* *ELOF1* *ACP5*
32	–	–	1	86047	C/G	0.297	−0.261	1.05e−05	0.046	*ACTN4* *ECH1*

Results of single-marker genome-wide association analysis using all birds. Candidate genes include protein-coding genes located within 5000 bp upstream or downstream of the QTL start or end site, respectively.

**Table 4:** Results of Bayesian multi-marker regression for wooden breast and white striping.

Trait	Chromosome	Window (Mb)	# SNPs	Explained genetic variance (%)	*p* > 0^1^
Wooden Breast	30	0 – 1	327	3.10	0.970
	5	14 – 15	186	2.74	0.808
	6	19 – 20	82	2.35	0.775
	6	23 – 24	990	1.72	0.951
	5	13 – 14	224	1.64	0.622
	5	16 – 17	436	1.60	0.798
	18	9 – 10	1508	1.44	0.987
	2	45 – 46	147	1.14	0.587
	18	4 – 5	791	1.01	0.890
White Striping	5	14 – 15	186	5.38	0.990
	5	58 – 59	604	3.18	0.997
	12	9 – 10	563	1.19	0.990

Windows identified by Bayes B were considered significant if they explained ≥ 1% of genetic variance. Body weight traits did not have any significant 1-Mb windows. P > 0 refers to the frequency of sample for which the region had a nonzero effect.

Top candidate genes for wooden breast (Table 3) include *cyclin dependent kinase inhibitor 1C* (*CDKN1C*), *cathepsin D* (*CTSD*), *potassium voltage-gated channel subfamily Q member 1* (*KCNQ1*), *lymphocyte-specific protein 1* (*LSP1*), *solute carrier family 22 member 18* (*SLC22A18*) and *USH1 protein network component harmonin* (*USH1C*) on GGA5 and *dynamin 2* (*DNM2*) on GGA30. In humans, variation in all of these genes has been linked to altered insulin expression or secretion^23–26^ despite their having distinct biological functions.

### GWAS results for white striping

**Figure 2:**
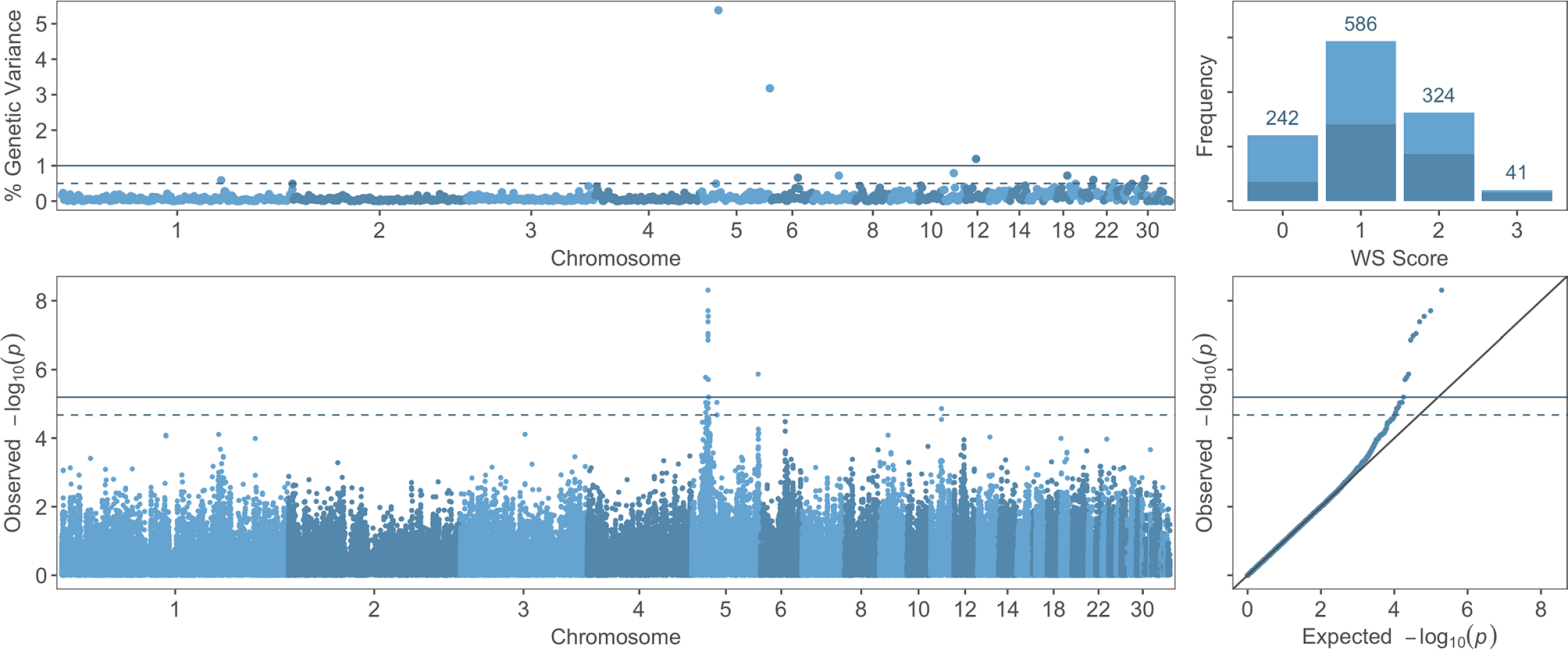
Genome-wide association results for white striping (WS) score using multi-marker (BayesB) and single-SNP (mixed linear model) analyses. (Top Left) Percentage of genetic variance explained by 1-Mb regions across the genome for WS score. (Top Right) Distribution of WS scores across progeny used in genome-wide association analyses; dark blue = male, light blue = female. (Bottom Left) Manhattan plot of single-SNP results showing the − log10(*p*-value) of SNPs ordered by chromosome and position. (Bottom Right) Quantile–quantile plot of p-values from single-SNP results of WS score. Solid and dashed lines indicate significant and suggestive thresholds, respectively, for each model.

A total of 18 SNPs (10 significant and 8 suggestive) were associated with white striping score (Fig. 2; Supplementary Table S7), all of which are located on GGA5, except for one marker on GGA11. Similar to the wooden breast association analysis results, most of these loci (9 significant and 6 suggestive, 83% of combined) were on GGA5, between 12.1 and 14.8 Mb (Table 5). Bayesian multi-marker regression identified a window in this same region, between 14.0 and 15.0 Mb, as the highest-ranking 1-Mb window, explaining 5.4% of genetic variance (Table 4). Two other windows also explain more than 1% of genetic variance, one on the long arm of GGA5 and one on GGA12. Together, the three significant windows for white striping explained 9.8% of the genetic variance for the trait. None of the QTLs identified in this study overlapped with QTLs previously identified for white striping; Pampouille et al.^19^ did not discover QTLs with genome-wide significance, but identified three QTLs with chromosome-wide significance in the 3rd Mb windows of GGA17 and GGA18 and in the 88th Mb window of GGA1. No significant or suggestive SNPs were identified for sex-specific analyses of white striping. Genomic windows with suggestive significance in the BayesB analyses are listed in Supplementary Table S6.

**Table 5:** White Striping QTL. Results of single-marker genome-wide association analysis using all progeny.

QTL				Top SNP in QTL						
Chromosome	Start (bp)	End (bp)	Number of significant SNPs	Position (bp)	Alleles (Effect/ Alternative)	Effect allele frequency	Effect size estimate	*p*-value	FDR	Candidate genes
5	–	–	1	12196653	T/C	0.232	− 0.239	1.68e−06	0.036	*USH1C*
5	14280580	14320311	4	14280613	T/C	0.335	0.228	9.07e−08	0.003	*LSP1* *TNNI2* *SYT8* *ENSGALG00000006608* *CTSD*
5	14321077	14321102	2	14321077	G/A	0.433	0.230	4.93e−09	0.001	*ENSGALG00000006608* *CTSD*
5	–	–	1	14477401	T/C	0.238	− 0.241	2.86e−08	0.002	*ENSGALG00000006608* *MOB2*
5	–	–	1	14529930	T/C	0.286	0.229	1.95e−06	0.038	*ENSGALG00000006608* *BRSK1*
5	–	–	1	58074636	A/G	0.359	0.166	1.35e−06	0.033	*NIN*

Candidate genes include protein-coding genes located within 5000 bp upstream or downstream of the QTL start or end site, respectively.

Top candidate genes for white striping include *CTSD*, *LSP1*, *troponin I2 fast skeletal type* (*TNNI2*), *synaptotagmin 8* (*SYT8*), and *MOB kinase activator 2* (*MOB2*). As with candidate genes for wooden breast, variation in mammalian homologues of all of these genes is linked to altered insulin expression or secretion in pancreatic beta cells^25,27^. However, they possess additional functions that may be relevant to poultry myopathies, such as calcium-dependent regulation of striated muscle contraction^28^ (*TNNI2*), and regulation neutrophil transendothelial migration^29^ (*LSP1*).

### GWAS results for body weight traits

No QTLs were identified for either body weight trait using single-SNP or multi-marker analyses (Supplementary Figs. S1 and S2; Supplementary Table S6). This may be a result extreme selection for growth rate over many decades, likely resulting in fixed alleles at loci of large effect. By contrast, wooden breast and white striping have a much shorter selection history based on their somewhat recent appearance, and have potentially been subjected to both positive and negative selection if they indeed share a strong genetic basis with performance traits such as feed efficiency and breast muscle yield. This would preserve variation at loci with moderate or large effects, which are easier to detect with lower sample sizes in genome-wide association analyses.

QTL detection for both body weight and muscle disorder traits may also have been hindered by the extremely rapid LD decay (Supplementary Table S2) in our 4-way crossbred broiler population relative to the genome coverage achieved by our genotyping methods and filter criteria. Using SNP positions and D_0.2_, we estimated that only 27.52% of autosomes and the Z chromosome were encompassed by SNPs in this study. This is a conservative estimate, as it includes chromosomal regions with extremely low variation, but indicates the strong potential for additional QTLs with large effects that were not captured by this study. Our sample size also prevented detection of small-effect QTLs by limiting statistical power. The rapid LD decay, however, gives us confidence that the QTLs detected in this study are in close proximity to their causal mutations.

### Comparative genomics of GGA5

Chicken chromosome 5 (GGA5) has conserved synteny with portions of human chromosomes 11, 14, and 15^30^. The majority of QTLs identified for both wooden breast and white striping were located in a region that has highly conserved synteny with HSA11 (Fig. 4). Indeed, there is greater conservation of synteny for genes on HSA11 between chickens and humans than between mice and humans, although numerous intrachromosomal rearrangements are present. The area on HSA11 that is homologous to QTL regions on GGA5 identified in the present study is associated with numerous metabolic and growth disorders and various forms of diabetes mellitus (Fig. 4). For example, *KCNQ1* locus in this region is heavily studied in humans as part of an imprinted gene cluster that when dysregulated can cause overgrowth disorders including Beckwith-Wiedemann syndrome^31^. Even though the size of the region and order of genes is nearly identical between chickens and mammals (Fig. 4), chickens lack the relevant imprinting control elements present in mammals^32^. This suggests that chickens may serve as an excellent model for studying topics such as evolutionary genomics, imprinting control, and growth disorders.

The QTL-rich region of GGA5 also contains several areas that show evidence of strong selection sweeps (Fig. 4) in two commercial purebred male broiler lines selected for breast muscle growth and feed efficiency^33^. Selective breeding for meat production traits is considered a major culprit for the rapid increase in meat quality issues among broiler chickens, and the genomic co-localization of loci that are beneficial for production traits with loci associated with breast muscle myopathies provides further evidence of this. Targeted sequencing of GGA5 is required to determine if the specific genetic mutations contributing to each trait are simply linked or, in fact, the same. Additionally, targeted sequencing of GGA5 with long-read technologies could reveal copy number variants, which are not captured by SNP data. The *SRY-box transcription factor 6* (*SOX6*) gene is of particular interest because it was identified as a candidate gene for the selection sweep region on GGA5^33^. Although *SOX6* was not identified as a candidate gene for wooden breast based on association analyses described here, a selection sweep in or near this gene would hinder detection of causal variants using many GWAS methods due to filters for minor allele frequency.

### Clinical significance of candidate genes

**Figure 3:**
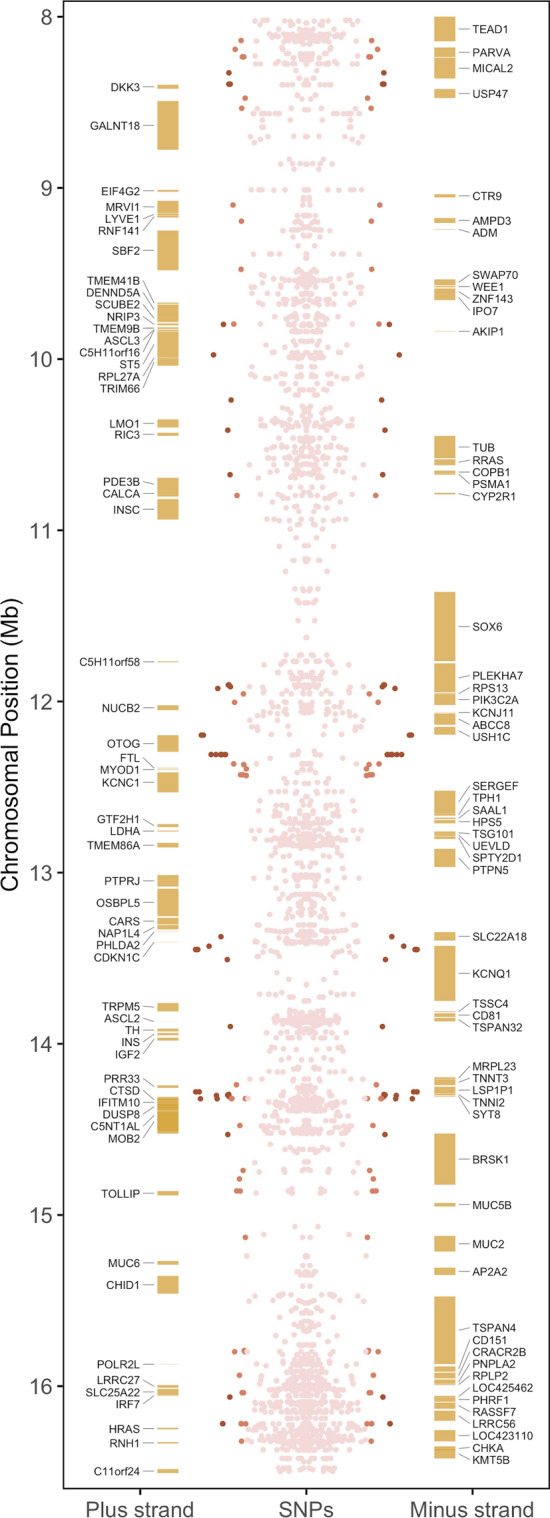
Gene landscape of QTL-rich region between 8.0 Mb and 16.5 Mb on chicken chromosome 5. Mirrored Manhattan plot shows SNPs with significance thresholds indicated by color; dark red = significant, orange = suggestive.

**Figure 4:**
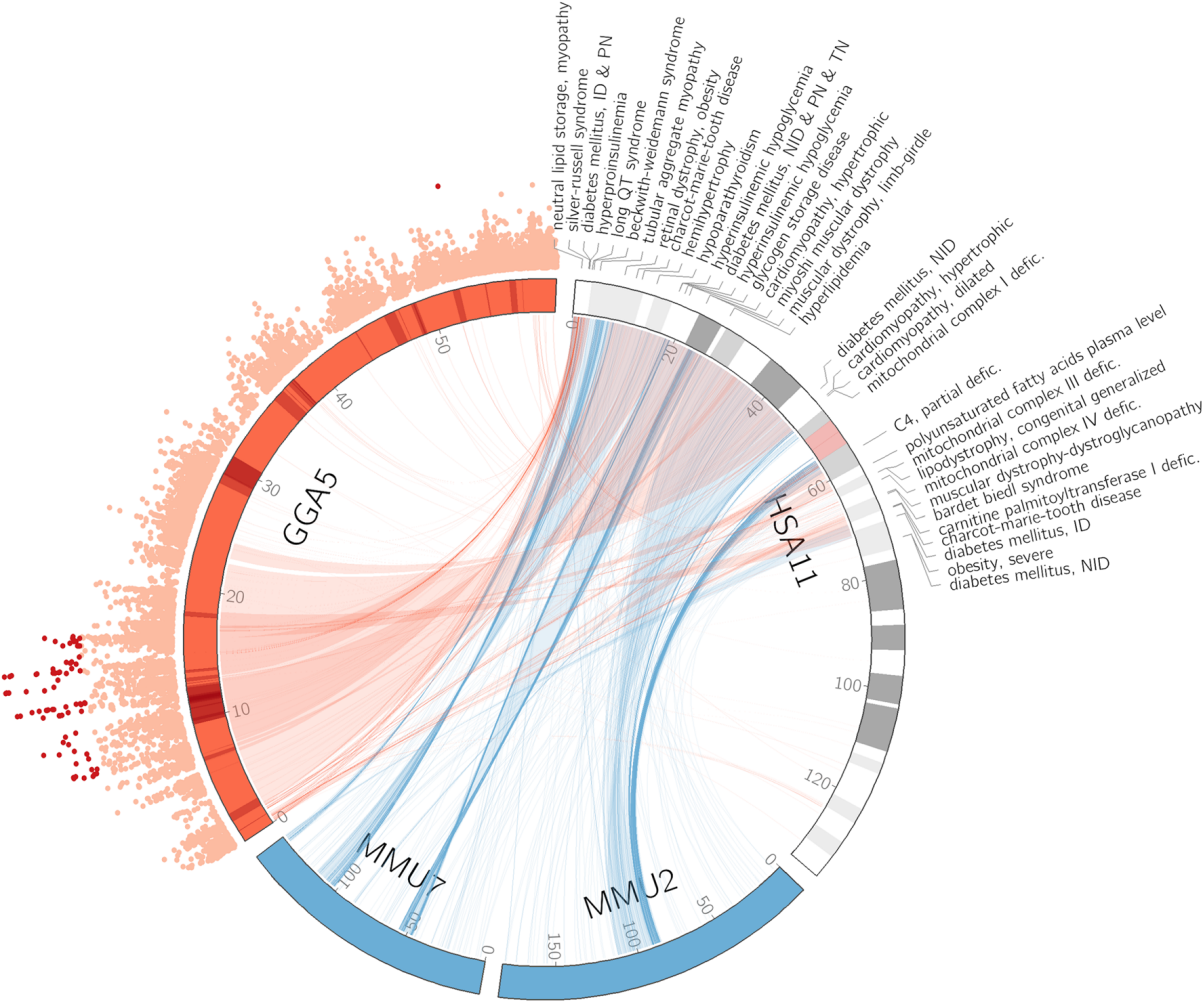
Conserved synteny between chickens and humans. The wooden breast QTL-rich region of chicken chromosome 5 (GGA5) has highly conserved synteny with human chromosome 11 (HSA11), especially compared to homologous regions in the mouse genome on chromosomes 2 (MMU2) and 7 (MMU7). In humans, this area is associated with a high number of growth and metabolic disorders, which are highlighted here based on existing knowledge of wooden breast and white striping, including a hypothesis suggesting dysregulation of lipid and glucose metabolism as an important underlying factor in development of the myopathies. The scatter plot on GGA5 represents GWAS results from the present study with significant SNPs in red. Red bands on GGA5 indicate previously identified selective sweeps in commercial purebred broiler lines that are susceptible to wooden breast with overlapping selective sweep regions in dark red. The centromere (pink) and G-banding (grey and white) are shown on HSA11 to improve visualization. Defic: deficiency, ID: insulin-dependent, NID: non-insulin-dependent, PN: permanent neonatal, TN: transient neonatal.

A major functional theme of candidate genes identified in this study is insulin secretion and action, particularly in the QTL-rich region of GGA5 (Fig. 3), which contains numerous genes previously shown to be associated with metabolic and cardiac disorders in humans (Fig. 4). For example, *KCNQ1* polymorphisms are associated with altered insulin secretion in pancreatic β cells, short and long QT syndrome in cardiac muscle, and sensorineural deafness in the inner ear^34^. This gene encodes a highly-studied voltage-gated potassium channel, which interacts with numerous other protein subunits and switches from voltage-dependent to constitutive activity^34^, endowing it with a diverse set of functional roles and associating it with a proportionally diverse number of pathologies. Some pleiotropic *KCNQ1* variants in humans can simultaneously increase insulin secretion in the pancreas, reduce serum potassium upon oral glucose challenge, and cause long QT syndrome, putting individuals at risk of sudden, uncontrollable, arrhythmias which may lead to fainting or sudden death^35^. This could represent a potential cause of sudden cardiac death reported in broilers, especially those with wooden breast^36^. We re-examined data from a previous study of differential gene expression associated with wooden breast in 2-week-old female broilers^16^ and found that, although there was no differential expression of *KCNQ1* in the pectoralis major of 2-week-old birds, expression levels of the gene were very low in all birds (data not shown). It is therefore likely that genetic variation in *KCNQ1* would primarily affect organs other than the pectoralis major, potentially including the pancreas.

Some candidate genes on GGA5 affect insulin secretion through both gene–gene interactions and protein function, as the insulin gene (*INS*) is located in the middle of this QTL-rich region at approximately 13.94 Mb (Fig. 3). For instance, *synaptotagmin 8* (*SYT8)* encodes a membrane protein with roles in trafficking and exocytosis, including insulin secretion in pancreatic islet cells^25^. The *SYT8* gene is located over 300 kb away from *INS* in humans, and yet physically interacts with the *INS* locus to elevate *SYT8* expression, especially in the presence of glucose^25^. This indicates the importance of *SYT8* for both basal and glucose-stimulated insulin secretion in human islets. The same study identified additional interactions of *INS* with *troponin I2, fast skeletal type* (*TNNI2*) and *lymphocyte-specific protein 1 (LSP1*), which are also top candidate genes for wooden breast and white striping.

QTLs outside of GGA5 have strong ties to insulin as well. The protein encoded by *DNM2* on GGA30 is critical for proper pancreatic function, where it regulates biphasic insulin secretion and glucose homeostasis in mammals. Knockout of *DNM2* in pancreatic β cells in mice causes glucose intolerance via remodeling of the actin cytoskeleton and inefficient endocytosis-exocytosis coupling, indicating a potential pathophysiological link between *DNM2* function and diabetes mellitus^24^. *DNM2* is best known for its involvement in two distinct congenital neuromuscular diseases of humans, centronuclear myopathy and Charcot-Marie-Tooth neuropathy. Several of the characteristic features of DNM2-associated centronuclear myopathy are reminiscent of the histopathological signs of wooden breast and white striping in broiler breast muscle, including increased variability of myofiber size, endomysial fibrosis, calcium homeostasis alterations, and abnormal centralization of nuclei in muscle fibers, which can also be seen in regenerating myofibers^37,38^. Although not discussed by Mutryn et al.^8^, *DNM2* expression was found to be downregulated in wooden breast-affected broilers compared to unaffected broilers at market age (data not shown), which is of particular interest considering findings of a knockout study of this gene in mouse gastrocnemius muscle. Tinelli et al.^39^ found that *DNM2* knockout caused a decrease in the number of muscle fibers, an increase in the proportion of smaller muscle fibers, an increase in lipid droplets, mitochondrial enlargement and disruption of cristae, mitochondrial dysfunction, and altered neuromuscular junctions at various developmental ages^39^. Previously, Papah et al.^5^ described an increase in acellular lipid droplets and abnormal mitochondrial morphology with degenerated cristae as part of the wooden breast pathogenesis. A decrease in the number of muscle fibers and increase in the proportion of small fibers has also been documented as a characteristic of wooden breast in some broiler lines^40^.

A candidate gene on GGA6, *GOT1,* encodes the cytoplasmic version of an enzyme called aspartate transaminase (AST) that catalyzes the interconversion of aspartate and α-ketoglutarate to oxaloacetate and glutamate. Two key roles of AST should be highlighted from this anaplerotic reaction: the replenishment of aspartate, a key intermediate of the citric acid cycle, and the regulation of concentrations of glutamate, which functions as a potentiator of insulin release and precursor of the antioxidant glutathione in human skeletal muscle^41^. Thus, variation in *GOT1* may play an important role in altered energy metabolism and ROS balance in wooden breast.

Genetic variation associated with broiler production traits such as visceral adiposity^42^, body weight^43^ is already known to affect plasma insulin and glucagon levels, as well as insulin sensitivity and glucose clearance. More generally, commercial broilers have elevated plasma insulin levels compared to layers^44^ and poorer serum insulin homeostasis compared to Silky chickens^45^. Insulin is also a key regulator of carbohydrate, lipid, and amino acid metabolism^46,47^, which are dysregulated in wooden breast and white striping^48,49^, although additional research is required to determine whether altered insulin dynamics are a contributing factor to wooden breast and white striping in broilers.

## Conclusions

This study is the first to characterize the genetic basis of wooden breast and white striping in a commercial crossbred broiler population using genetic markers. A major finding of the present work is the identification of a QTL-rich region for both wooden breast and white striping between 8.0 and 16.5 Mb on GGA5, which is homologous to and possesses highly conserved synteny with an imprinted region of human chromosome 11. Further study of this GGA5 region may prove to be critically important for understanding the cause of chicken myopathies as well as the evolution of genomic imprinting and genes involved in growth regulation in humans. Our findings also provide compelling evidence in support of a previous hypothesis describing a shared pathomechanism between breast muscle myopathies in broilers and type 2 diabetes in mammals^15,16^ and suggest that potential alterations to pancreatic function and insulin action may be involved. There exist substantial differences between mammalian and avian insulin signaling and action—for example, the apparent lack of the insulin responsive glucose transporter *GLUT4* in the chicken genome^50^—that warrant additional research, especially with regard to wooden breast and white striping. Investigation into organs involved in regulation of whole-body energy homeostasis, such as the pancreas, liver, and adipose tissue, may also aid in our understanding of these myopathies.

## Methods

### Birds

All animal procedures were performed in accordance with guidelines set by The University of Delaware Institutional Animal Care and Use Committee (IACUC) and were approved by IACUC under protocol number 48R-2015-0. The study was carried out in compliance with the ARRIVE guidelines. A total of 1,228 mixed male and female Cobb500 broilers from the same breeding population of 15 sires and 200 dams were raised as two separate hatches (n_1_ = 686, n_2_ = 542) with staggered hatch dates. Broilers were housed according to optimal industry standards in five poultry houses and given free access to feed and water until approximately 7 weeks of age (specifically 48, 49, 52, or 53 days), at which time they were euthanized by cervical dislocation. Live weight was recorded for all birds at 13 days of age. Preceding euthanasia (specifically 47, 48, or 52 days of age), live weight was recorded again and whole blood samples were collected from the brachial wing vein of each bird using a 3 mL syringe with 23-gauge needle and placed in lithium heparin-coated tubes. Plasma was separated by centrifugation and blood samples were stored at -80˚C until further analysis.

During necropsy, the pectoralis major muscles were evaluated for gross lesions and palpable firmness associated with wooden breast and each bird was assigned a wooden breast score using a 0–4 scale; 0-Normal indicates the bird had no macroscopic signs of the myopathy, 1-Very Mild indicates approximately 1% or less of the breast muscle was affected, 2-Mild indicates approximately between 1 and 10% of the breast muscle was affected, 3-Moderate indicates approximately between 10 and 50% of the breast muscle was affected, and a score of 4-Severe indicates that more than 50% was affected. This scoring system was employed by Lake et al.^16^ in order to separate unaffected, mildly, and moderately affected chickens with higher resolution and greater sensitivity. White striping was also assessed at this time and each bird was assigned a white striping score using a 0–3 scale; 0-Normal indicates the bird had no macroscopic signs of white striping, 1-Mild indicates approximately 20% or less of the breast muscle was affected, 2-Moderate indicates approximately between 20 and 50% of the breast muscle was affected, and a score of 3-Severe indicates more than 50% of the muscle was affected. The incidence rates of wooden breast and white striping in males and females was compared using Pearson's Chi-squared test implemented in R with Yates' continuity correction.

### GBS library construction and DNA sequencing

After the live animal experiment was completed, blood samples from 1194 birds were selected for DNA extraction and genotyping. These represented all males (n = 557) and females (n = 636) that did not exhibit any noticeable health conditions or deformations and had a blood sample of sufficient size with no sign of coagulation. The 15 sires of our chicken population were also included for DNA extraction and genotyping to improve genotype calling of progeny; however, phenotypic data was not recorded for sires. Total DNA was isolated from blood samples using the DNeasy Blood and Tissue Kit (Qiagen) according to the manufacturer’s protocol. DNA samples were quantified and quality was assessed using the NanoDrop 1000 Spectrophotometer (Thermo Fisher scientific); all samples had a 260/280 ratio of approximately 1.8 and 260/230 ratio greater than 1.5. Approximately 2.5 µg of DNA per sample was aliquoted into individual wells of a 96-well plate, dried at room temperature, sealed, and shipped to Animal Genomics Research laboratory, AgResearch Invermay, New Zealand for restriction enzyme-based genotyping by sequencing (GBS). The GBS libraries were constructed according to the methods outlined in Elshire et al.^51^ with modifications as in Dodds et al.^52^ using two restriction enzymes (*MspI* and *ApeKI*). Single-end 1 × 100 sequencing of 96-plex libraries was performed on an Illumina HiSeq 2500. To control and detect lane bias and batch bias, three samples of control DNA were included in each library and each library was run on at least 4 lanes across at least 2 flowcells. Additionally, one sample (B92023) was included as a positive control in all libraries.

### Data filtering and SNP calling

AgResearch supplied a single variant call file (VCF) which was constructed by the following methods. Fastq files were demultiplexed using GBSX^53^ and mapped by sample and lane onto the GRCg6a reference genome (Ensembl release 95) using BWA-MEM^54^. Samtools v1.9^55^ was used to remove reads with mapping quality below 30, convert the alignment file to BAM format, and merge all alignment files by sample. Variants were detected across all samples on a per chromosome basis using samtools and bcftools v1.9 with parameters set to limit variants to biallelic SNPs^55^. The results were then combined into a single VCF.

Variants were filtered to only include SNPs with a minimum read depth of 5 for at least 50% of samples, a maximum average read depth of 50, and a minor allele frequency of 5% or greater. Samples were also filtered to only include those with a minimum read depth of 5 for at least 50% of SNPs. Loci on the W chromosome were excluded from all analyses, but were used along with Z chromosome loci to confirm sex of the birds before subsequent analyses. Loci on the Z chromosome were filtered separately using only males to avoid filtering bias from hemizygotic females.

### Relatedness and genetic parameters

A genomic relationship matrix (***G***) was constructed from the resulting variant data using the R package KGD^52^, which implements a method developed to account for GBS with low depth of coverage. Variance components and heritabilities of wooden breast, white striping, body weight at 13 days, and body weight at 7 weeks were estimated using ASReml 4^56^ with the following univariate model:

1 $${\varvec{y}} = {\varvec{Xb}} + {\varvec{Zu}} + e,$$

where ${\varvec{y}}$ is a vector of phenotype values for the relevant trait, ${\varvec{b}}$ is the vector of fixed effects and the overall mean (a vector of 1s), ${\varvec{X}}$ is an incidence matrix for fixed effects, ${\varvec{u}}$ is a vector of random polygenic effects, ${\varvec{Z}}$ is an incidence matrix corresponding to ${\varvec{u}}$, and $e$ is the residual error. Fixed effects included sex, poultry house, and age at necropsy for the phenotypes of wooden breast score and white striping score. For analysis of body weight, only sex and poultry house were included as fixed effects; although live weight at 7 weeks was measured on three separate days, this effect was disregarded because it was perfectly correlated with poultry house. Random effects $u$ and $e$ are assumed to follow normal distributions: ${\varvec{u}}\sim N\left(0,{\sigma }_{g}^{2}{\varvec{G}}\right)$ and $e\sim N\left(0,{\sigma }_{e}^{2}{{\varvec{I}}}_{n}\right)$ where ${\sigma }_{g}^{2}$ is the genetic variance, ${\sigma }_{e}^{2}$ is the variance of the residual errors, ${\varvec{G}}$ is the genomic relationship matrix as defined above, and ${{\varvec{I}}}_{n}$ is an identity matrix of dimension $n$. Heritability was calculated as the ratio of genetic to phenotypic variance, i.e. the sum of genetic and residual variance. Pairwise genetic and phenotypic correlations between traits were estimated using bivariate models with the same fixed and random effects as specified for the univariate analyses.

### Linkage disequilibrium analysis

Pairwise linkage disequilibrium (LD; r^2^) of markers within 5 Mb of each other was calculated with Haploview v4.2 using the discrete genotype calls produced during the SNP calling step described above. To characterize LD decay with distance, markers were filtered to only include SNPs with a minimum read depth of 10 in at least 50% of birds. LD on the Z chromosome was determined using genotype calls from only male birds. LD decay curves were generated for macro-chromosomes (GGA1 through GGA5), intermediate chromosomes (GGA6 through GGA10), micro-chromosomes (GGA11 through GGA33), and the Z chromosome by fitting a four-parameter Weibull function (type-1) to r^2^ values against physical distance using the R package ‘drc’ (v3.0–1). The resulting curves were used to estimate r^2^ at distances of 1 kb, 10 kb, 100 kb, 500 kb, 1 Mb, and 5 Mb, and to calculate the distance at which LD decayed below the threshold of 0.2 (D_0.2_).

The ability of the present study to capture QTL was evaluated by examining SNP coverage on each chromosome in comparison to corresponding D_0.2_ estimates. Specifically, we calculated the percentage of each chromosome in close enough proximity to a SNP for LD to be considered useful (i.e. D_0.2_), then computed the average percentage weighted by chromosome length. This final value reflects the percentage of the genome (autosomes and Z chromosome) that was captured by SNPs in the present dataset.

### Allele dosage estimation

Due to the relatively low-depth sequencing methods implemented here, continuous genotype probabilities were used for all association analyses rather than discrete genotype calls. Genotype posterior probabilities were calculated using the Bayesian genotype caller polyRAD v1.1^57^, with population structure and LD used as a prior and default parameter values. The minimum correlation coefficient between two alleles (r^2^) for LD to be used in genotype estimation was set at 0.2 and the distance within which to search for alleles that may be in LD with the given allele was set to 10,000 base pairs. The maximum mean difference in allele frequencies between iterations to be tolerated before iterations end was set to 0.001. The resulting genotype posterior probabilities were converted to allele dosages (i.e. posterior mean genotypes) for use in association analysis. Genotype calling of markers on the Z chromosome was performed using only male birds.

### Genome-wide association analyses

Two separate approaches—Bayesian multi-marker regression and single-SNP analysis—were used to detect quantitative trait loci (QTL) for each trait. Bayesian models were implemented in GenSel^58^ and involved first estimating the proportion of markers with null effect (π) using BayesCπ, followed by computation of the genetic variance explained by each 1 Mb window of SNPs in the genome with BayesB. The BayesCπ algorithm was run for 200,000 MCMC iterations with a burn-in of 150,000 iterations and used a starting value of 0.5 for π. BayesB was run for 60,000 iterations with a burn-in of 20,000 and used the posterior mean of π calculated by BayesCπ for each trait. The following model was used for both Bayesian methods:

2 $${\varvec{y}} = {\varvec{Xb}} + \mathop \sum \limits_{k = 1}^{K} {\varvec{z}}_{k} a_{k} + e,$$

where $K$ is the number of SNPs, ${z}_{k}$ is a vector of allele dosages at SNP $k$, ${a}_{k}$ is the additive effect of that SNP, and the remaining variables are as previously described. Loci on the Z chromosome were disregarded for this analysis. Windows explaining greater than 1% of genetic variation were considered significant and those explaining greater than 0.5% were considered suggestive.

Single-SNP analysis was performed with a mixed linear model using GCTA version 1.26.0^59^ with software patches provided by van den Berg et al.^60^ that adapted the program to accept allele dosages. Equation 1 was used for all single-SNP analyses with the addition of a SNP effect fitted as a fixed covariate. Single-SNP analysis was performed three separate times for each trait, once using all birds, once with only females, and once with only males. Loci on the Z chromosome were only included when male birds were analyzed separately. For analyses including only male or female progeny, the fixed effect of sex was dropped from the model. Significant and suggestive genome-wide association thresholds were set at p-values corresponding to FDR-adjusted^61^ q-values of 0.05 and 0.20, respectively.

Significant intra-chromosomal SNPs that were in high LD (r^2^ > 0.75) or that were separated by a distance less than or equal to the D_0.2_ value for the relevant chromosome size were assumed to share evidence in association analyses for a given trait and were thus grouped into non-overlapping QTL. Markers considered “suggestive” were not used in the construction of QTL. Candidate genes were identified based on proximity to QTL (within 5000 bp up- or downstream of the start or end position of a QTL, respectively) using Ensembl and NCBI annotation of the *Gallus gallus* genome version GRCg6a (annotation release 99).

### Comparative genomics

An ~ 8 Mb region of GGA5 was found to be exceedingly rich in QTL for both wooden breast and white striping, and warranted additional attention with regard to existing knowledge of the chromosome’s genomic features, which were visualized in a Circos plot^62^. To this end, sequence alignments between chicken genome assembly GRCg6a and human genome assembly GRCh38 (vertebrate net alignment netHg38) and between human genome assembly GRCh38 and mouse genome assembly GRCm38 (placental net alignment netMm10) were retrieved using the UCSC Table Browser^63^. Associations with human muscular and metabolic disease phenotypes in genomic regions homologous to the QTL-rich section of GGA5 were identified from the OMIM database^64^. Additionally, previously identified selective sweeps on GGA5 in commercial purebred broiler lines that are susceptible to wooden breast^33^ were lifted over to the GRCg6a chicken genome assembly using the UCSC liftOver tool^65^.

## Supplementary Information

Supplementary Information 1.

Supplementary Information 2.

Supplementary Information 3.

Supplementary Information 4.

Supplementary Information 5.

Supplementary Information 6.

Supplementary Information 7.

Supplementary Information 8.

## Data Availability

The datasets generated during the current study are available in the NCBI Sequence Read Archive (SRA) repository under BioProject PRJNA682423.
