# Supplementary material for: Genetic basis and identification of candidate genes for wooden breast and white striping in commercial broiler chickens
Source: Sci Rep. 2021 Mar 24;11:6785. doi: 10.1038/s41598-021-86176-4 (PMC7990949; doi:10.1038/s41598-021-86176-4)
Supplement: Supplementary file 8 — Supplementary Information 8. [file 41598_2021_86176_MOESM8_ESM.docx]

Genetic basis and identification of candidate genes for wooden breast and white striping in commercial broiler chickens

Juniper A. Lake^1,2^, Jack C.M. Dekkers^3^, Behnam Abasht^1,2*^

^1^ Center for Bioinformatics and Computational Biology, University of Delaware, Newark, DE, USA

^2^ Department of Animal and Food Sciences, University of Delaware, Newark, DE, USA

^3^ Department of Animal Science, Iowa State University, Ames, IA, USA

^*^ Corresponding author

E-mail: abasht@udel.edu (BA)


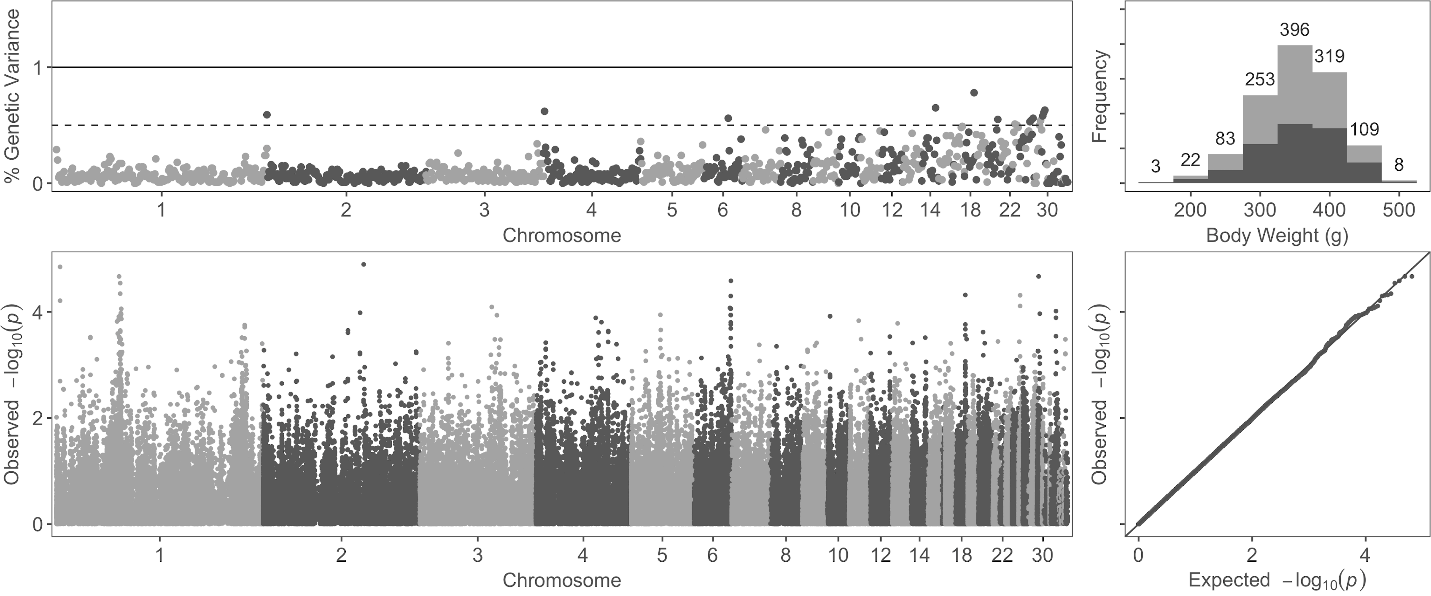


**Supplementary Figure S1.** Genome-wide association results for body weight at 13 days using multi-marker (BayesB) and single-SNP (mixed linear model) analyses. (Top Left) ﻿Percentage of genetic variance explained by 1-Mb regions across the genome for body weight at 13 days. Solid and dashed lines indicate significant (1%) and suggestive (0.5%) thresholds. (Top Right) Distribution of body weight at 13 days across progeny used in genome-wide association analyses; dark grey = male, light grey = female. (Bottom Left) ﻿Manhattan plot of single-SNP results showing the −log10(p-value) of SNPs ordered by chromosome and position. No markers pass the significant or suggestive thresholds. (Bottom Right) Quantile-quantile plot of p-values from single-SNP results of body weight at 13 days.


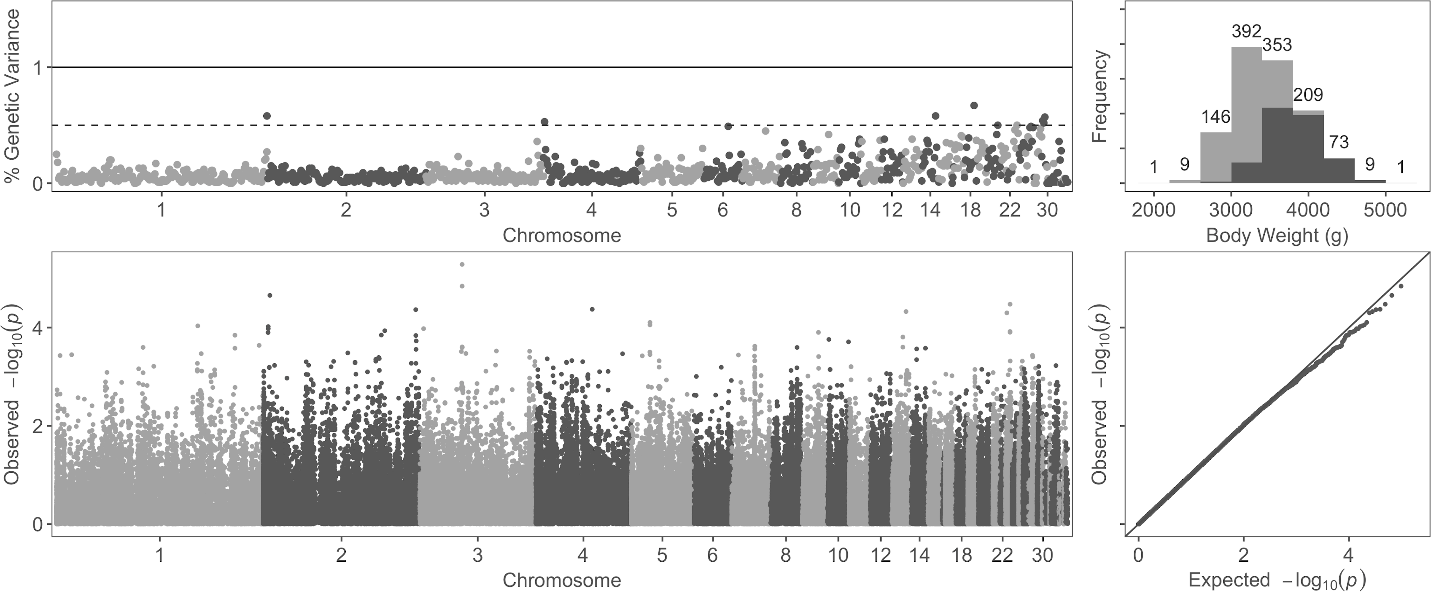


**Supplementary Figure S2.** Genome-wide association results for body weight at 7 weeks using multi-marker (BayesB) and single-SNP (mixed linear model) analyses. (Top Left) Percentage of genetic variance explained by 1-Mb regions across the genome for body weight at 7 weeks. Solid and dashed lines indicate significant (1%) and suggestive (0.5%) thresholds. (Top Right) Distribution of body weight at 7 weeks across progeny used in genome-wide association analyses; dark grey = male, light grey = female. (Bottom Left) ﻿Manhattan plot of single-SNP results showing the −log10(p-value) of SNPs ordered by chromosome and position. No markers pass the significant or suggestive thresholds. (Bottom Right) Quantile-quantile plot of p-values from single-SNP results of body weight at 7 weeks.
